# Multi-Stage Surgical Debulking for Advanced Lower Limb Lymphedema: Achieving Cosmetic and Functional Success

**DOI:** 10.7759/cureus.73053

**Published:** 2024-11-05

**Authors:** Odai G Bani Monia, Gaith I AlSaket, Ali M AlKadhimi, Ahmad M AlAzaideh, Bareqa I Salah

**Affiliations:** 1 Department of General Surgery, Jordan University Hospital, Amman, JOR

**Keywords:** charles procedure, lymphedema, quality of life, surgery, treatment

## Abstract

Lymphedema, characterized by impaired lymphatic drainage, presents in primary and secondary forms, causing limb enlargement and other complications. Management involves a multidisciplinary approach, with manual lymphatic drainage and surgery as key interventions. Treatment aims to improve quality of life, with surgical debulking showing positive outcomes, as demonstrated in a case of severe lower limb lymphedema.

A 40-year-old male with severe congenital lymphedema praecox presented with left lower extremity swelling and cellulitis. Despite previous unsuccessful surgery, subsequent debulking surgeries over nine months improved function and appearance. Biopsies confirmed lymphedema praecox diagnosis.

Lymphedema poses significant challenges, often requiring surgical intervention such as the Charles procedure, which involves surgically removing skin and soft tissue layers down to the deep fascia in the affected limb, with the excised skin repurposed as a graft for coverage, in severe cases. However, milder cases may go unnoticed, leading to delayed treatment. Our patient experienced advanced lymphedema, necessitating a staged surgical approach to minimize risks and enhance outcomes. This strategy successfully managed blood loss and improved cosmetic results, ultimately improving the patient's quality of life.

Lymphedema poses complex challenges, with tailored treatments such as staged procedures essential for optimal outcomes. Our case emphasizes the need for careful consideration and patient counseling, highlighting the value of strategic management approaches. By minimizing risks and optimizing outcomes, we aim to enhance the quality of life for individuals with lymphedema, underscoring our commitment to ongoing improvement in patient care.

## Introduction

Lymphedema, characterized by fluid accumulation due to impaired lymphatic drainage [1], presents in primary and secondary forms. Primary lymphedema, stemming from underdeveloped lymphatics at birth, includes early-onset (lymphedema praecox) and late-onset (lymphedema tarda) varieties. Secondary lymphedema arises from damage or obstruction to the lymphatic system, often linked to conditions such as neoplasia, its treatment, or chronic venous insufficiency, including past deep venous thrombosis [2]. The persistent accumulation of protein-rich lymphatic fluid triggers inflammation, leading to fibrosis and further lymphatic damage, exacerbating the condition [3]. This leads to significant limb enlargement, increased infection risk, mobility impairment, and psychosocial distress [4].

Managing lymphedema necessitates a multidisciplinary approach involving specialists such as lymphedema experts, vascular surgeons, physiotherapists, and dermatologists. While a definitive cure remains elusive, manual lymphatic drainage therapy or complete decongestive therapy administered by certified therapists serves as the gold standard [5]. Surgical interventions become imperative when conservative methods prove inadequate, aiming to reduce limb size, prevent infections, halt disease progression, and enhance function [6].

Treatment encompasses mechanical measures such as limb elevation, compression garments, manual drainage, and hygiene upkeep [2]. The objective is to alleviate swelling and associated complications, thereby improving patients' quality of life [7]. Treatment modalities range from conservative approaches to surgical options, with advanced procedures such as vascularized lymph node transfer and lympho-venous bypass gaining prominence, particularly in developed regions [8]. Surgical debulking for lymphedema has a long history, with techniques dating back to 1912, focusing on tissue excision to enhance function [9,10].

We present the case of severe lower limb lymphedema effectively managed through multi-stage debulking surgery, highlighting the procedure's favorable impact on patient well-being.

## Case presentation

**Figure 1:**
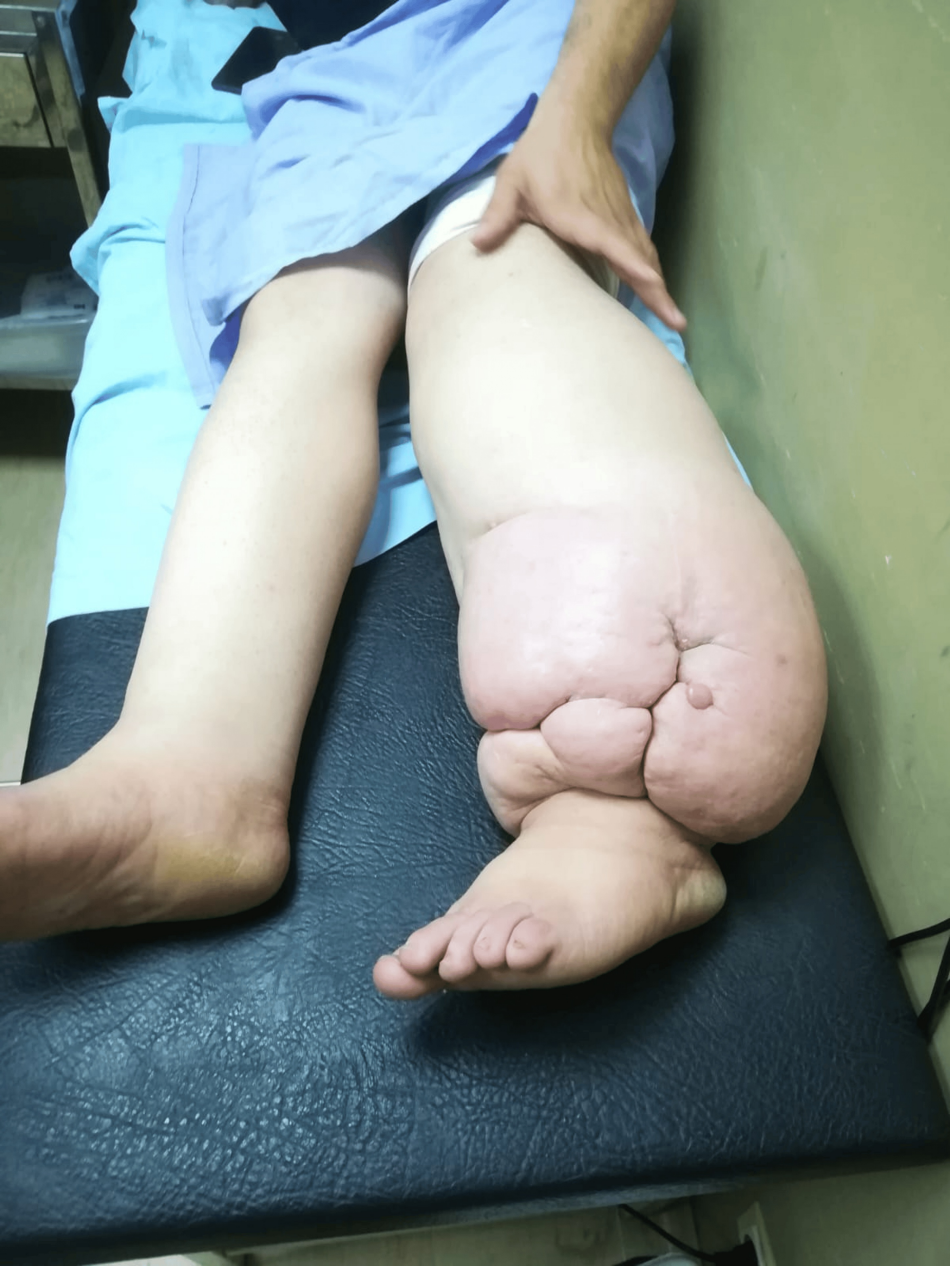
The patient’s left lower limb swelling and redness at the first presentation

**Figure 2:**
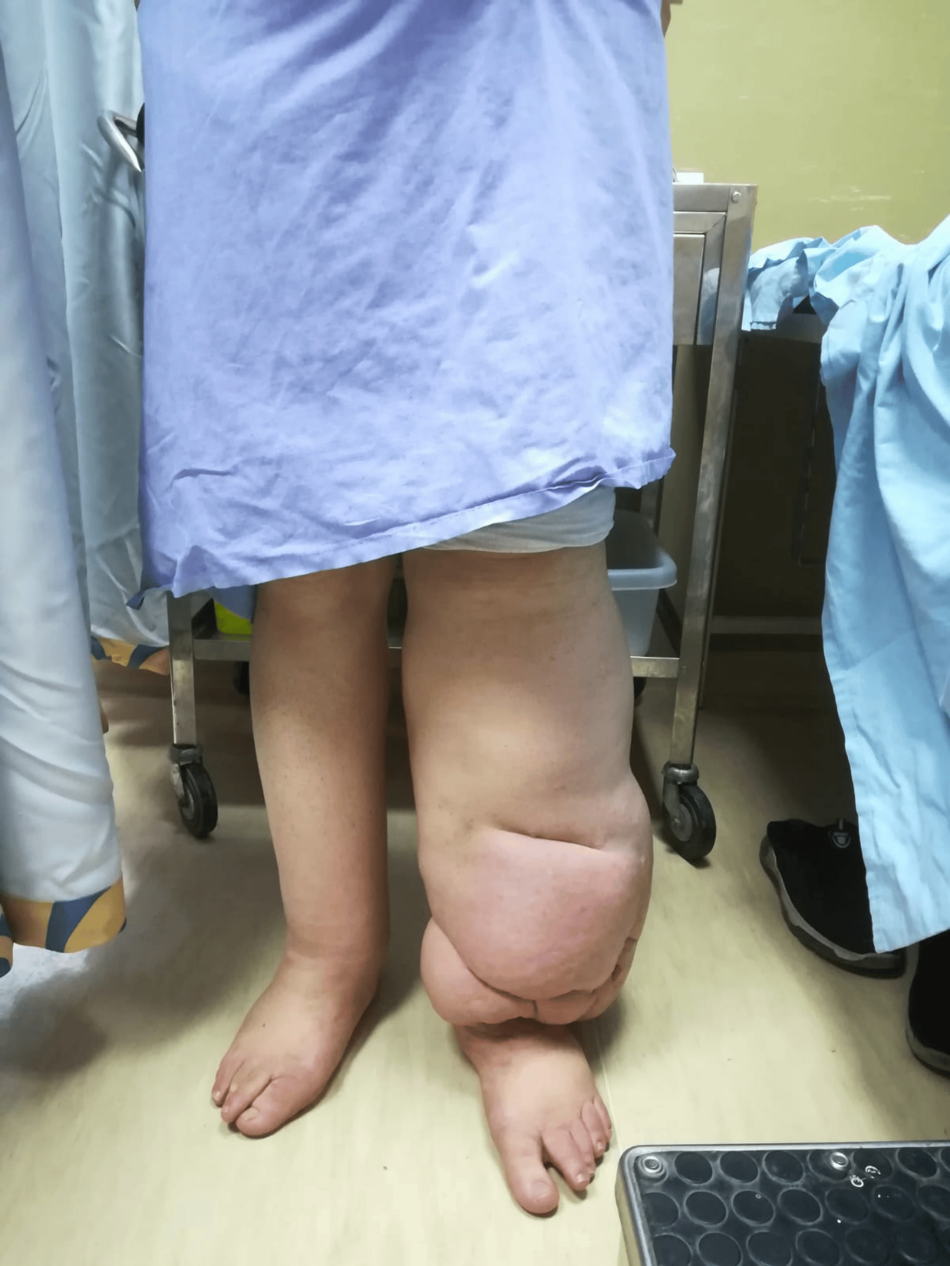
The patient’s left lower limb swelling compared to the right lower limb

A 40-year-old male government employee, medically unremarkable, with a history of severe congenital lymphedema presented to the outpatient clinic of the Plastic Surgery Department at Jordan University Hospital (JUH) with left lower extremity swelling, redness, and warmth, indicative of lymphedema praecox complicated by cellulitis (Figures 1, 2). On examination, the patient was alert and oriented. He was unable to bear weight on the affected limb. The physical examination showed palpable pulses in the dorsalis pedis, posterior tibial, and peroneal arteries, normal muscle strength (graded 5/5), and unremarkable neurological findings, prompting admission based on clinical assessment.

Further inquiry revealed a history of lymphedema since age 14, initially affecting the right lower extremity, which spontaneously resolved without intervention. However, the left-sided lymphedema persisted. There was no family history of the condition. The patient had previously undergone unsuccessful surgery for left-sided lymphedema at another hospital. Hospitalization was required for cellulitis treatment, which concluded without complications. Discharge instructions included oral antibiotics, pain relief medication, and limb elevation, with a follow-up outpatient appointment scheduled to discuss debulking procedures.

**Figure 3:**
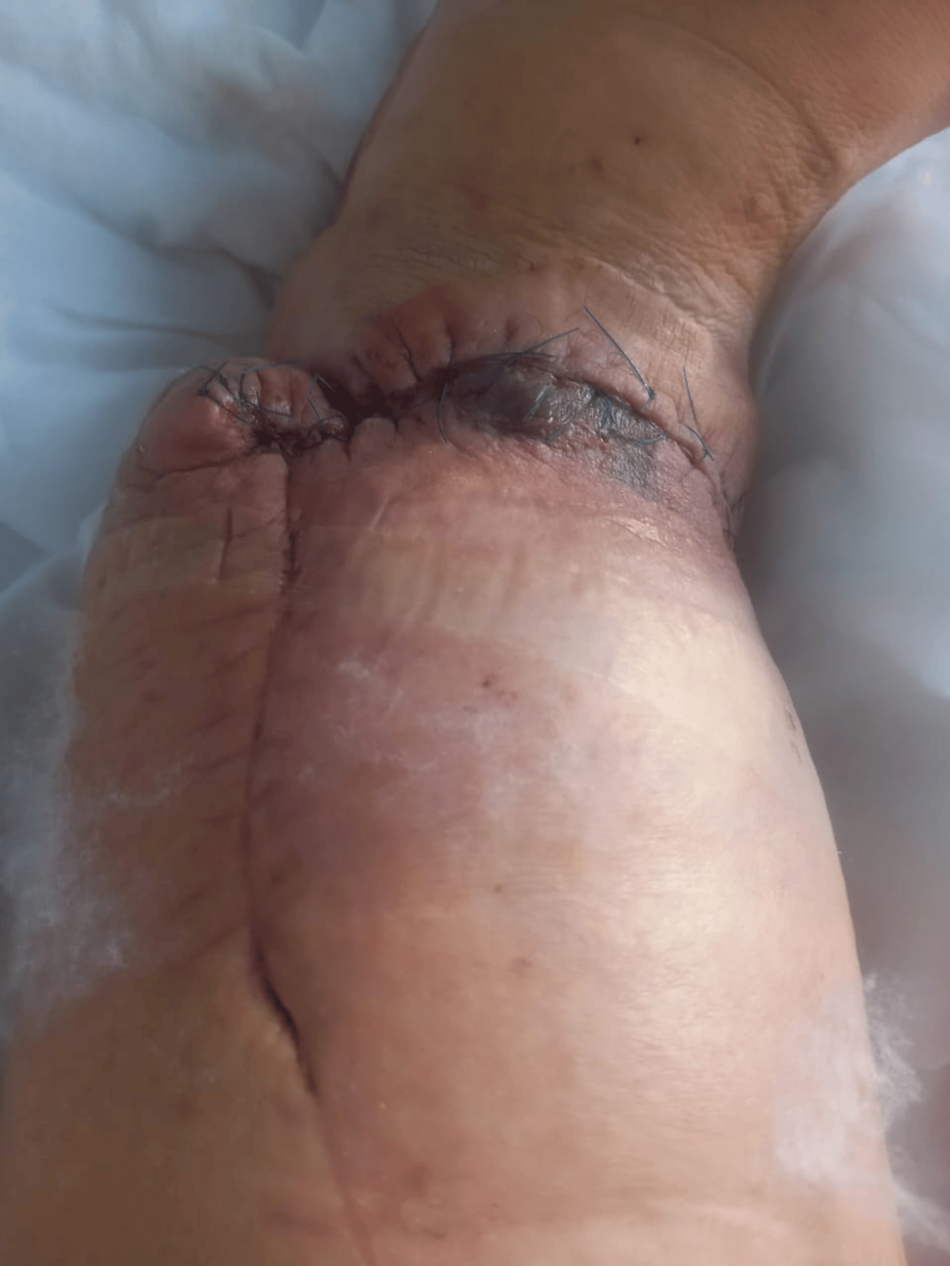
The patient’s left lower limb after the first debulking surgery

**Figure 4:**
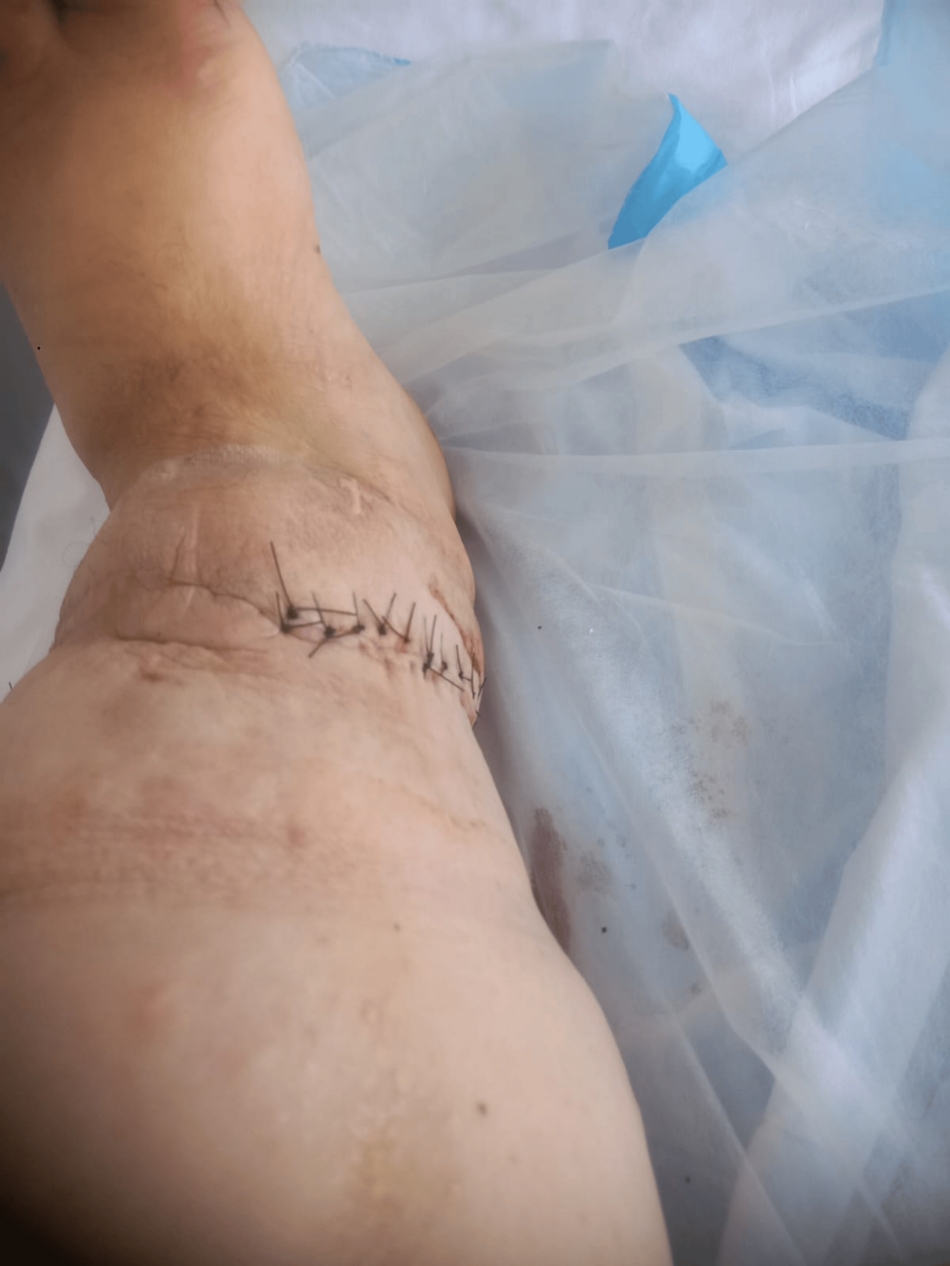
The patient’s left lower limb after the multiple debulking surgery

**Figure 5:**
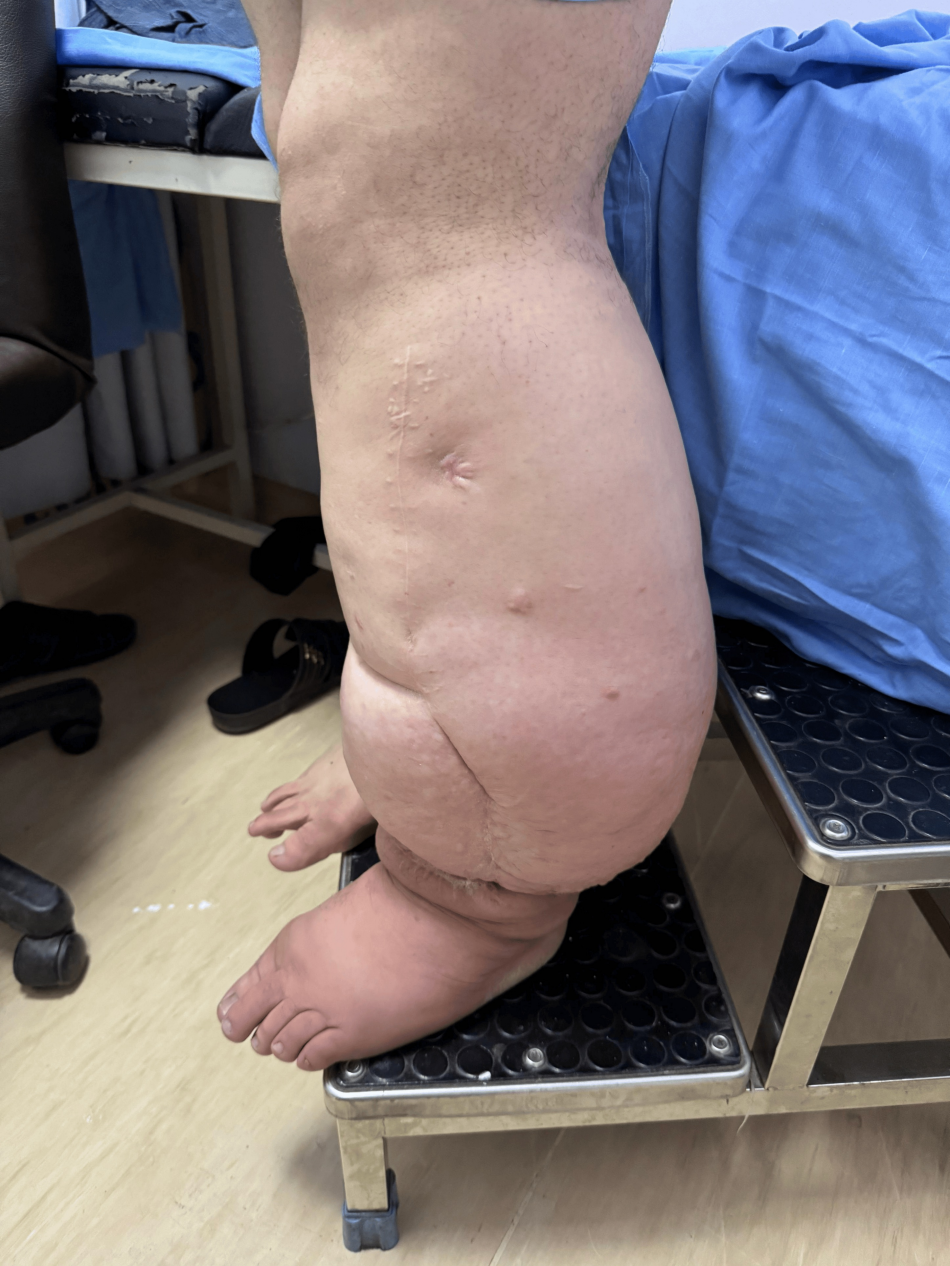
Healed vertical incision at follow-up after the ninth debulking procedure

During subsequent admissions, the patient underwent comprehensive diagnostic evaluations, including pre-operative laboratory tests such as complete blood count, kidney function test, coagulation profile, hemoglobin A1C, and urine analysis, as well as echocardiography and electrocardiography, all of which returned normal results. Over nine months, the patient underwent several debulking surgeries. These procedures involved making a longitudinal incision on the lateral aspect of the leg, creating superior and inferior flaps, excising lymphatic tissue, facilitating drainage and irrigation, applying hemostatic glue, and closing the skin using the mattress technique (Figure 3). Each excised lymphatic tissue sample underwent a biopsy, with all reports consistently indicating multiple fragments of fibrofatty tissue with intersecting fibrous bands and edema, without any signs of malignancy. This supported the diagnosis of lymphedema praecox. The consecutive surgeries resulted in significant functional and cosmetic enhancements, allowing the patient to resume daily activities and work with improved efficiency compared to his preoperative state (Figures 4, 5).

## Discussion

Lymphedema imposes a significant physical and emotional burden on those affected. Addressing this condition presents considerable complexities, with surgery typically being reserved for individuals with intricate needs unresponsive to conservative treatments [7]. Over the years, surgical techniques for lymphedema have evolved, with the Charles procedure, introduced in 1912, standing out as a prominent debulking method [9]. This approach involves surgically removing skin and soft tissues from the affected limb down to the deep fascia, utilizing the excised skin as a graft for coverage. However, milder cases of lymphedema often escape detection, resulting in insufficient attention to patients with less severe symptoms. Without timely intervention, lymphedema progresses to more severe stages, demanding more extensive resources [11].

This patient presented with advanced lymphedema severely impacting his daily life, hindering mobility, hygiene maintenance, and even clothing and footwear fitting. Given the prolonged history and significant swelling and functional impairment, conservative and physiological treatments commonly employed in developed nations were deemed inadequate. Although the Charles procedure has historically been the go-to option for such cases, its extensive nature poses risks such as substantial blood loss, infection, and prolonged hospital stays, necessitating thorough patient education, counseling, and discussion [6].

In this approach, we opted for a staged procedure to minimize blood loss, reduce anesthesia duration, and allow for adequate recovery between surgeries. Conducting a one-stage radical removal of extensive leg lymphedema can be arduous and may result in significant blood loss, particularly in settings with limited access to blood products. The staged approach aims to mitigate the risk of excessive blood loss, enhancing management effectiveness, particularly in resource-limited environments. In this case, the patient required only one unit of packed red blood cells during the procedure, indicating successful management of blood loss. Since our patient is young and has no chronic medical conditions, we did not experience any anesthesia-related complications during any of the procedures. Additionally, the debulking procedures were brief, lasting only one hour each, which alleviated concerns about anesthesia stress or complications for the patient, and this was reflected in our outcomes.

Furthermore, we avoided the need for a skin graft as there was ample viable tissue available to cover the defect, leading to an improved cosmetic outcome for the patient. Despite experiencing some postoperative pain and discomfort, which is common after surgical procedures, the patient expressed satisfaction with the results, markedly enhancing his quality of life post-surgery.

## Conclusions

In conclusion, lymphedema presents a multifaceted challenge, imposing both physical limitations and emotional distress on those affected. While surgical intervention, such as the Charles procedure, offers promise for individuals with severe lymphedema, its extensive nature and associated risks necessitate careful consideration and thorough patient counseling. This case underscores the importance of tailoring treatment approaches to individual patient needs and circumstances.

By adopting a staged procedure, we successfully minimized risks such as blood loss and optimized patient outcomes, particularly in resource-constrained settings. This experience highlights the value of a strategic approach to lymphedema management, aiming not only to alleviate physical symptoms but also to enhance the overall quality of life for patients. As we continue to refine our treatment strategies, we remain committed to addressing the diverse needs of individuals living with lymphedema, ultimately striving for improved patient outcomes and well-being.
